# Congenital Lobar Overinflation Presenting with Recurrent Pneumothoraces

**DOI:** 10.7759/cureus.5830

**Published:** 2019-10-03

**Authors:** Nina Hanžič, Urban Čizmarević, Matija Žerdin

**Affiliations:** 1 Radiology, University Medical Centre Maribor, Maribor, SVN

**Keywords:** congenital lobar overinflation, respiratory distress, pneumothorax, hyperinflation, neonate

## Abstract

Congenital lobar overinflation (CLO) is a rare neonatal developmental anomaly. We present the case of a newborn boy who was born as a first twin at the 26th gestation week, and transferred to the department of pediatric intensive care due to respiratory distress and prematurity. Recurrent pneumothoraces were observed, and the chest drain was inserted. However, saturation was unsatisfactory although he was intubated and ventilated by synchronized intermittent-mandatory ventilation (SIMV). The chest radiographs showed hyperinflation of the right middle lobe, with mediastinal shift to the left, and atelectasis of the remaining lobes. That caused ventilation and perfusion mismatch and hypoxia, due to a decrease in ventilated lung tissue. When high-frequency ventilation was instituted, he stabilized. Computed tomography (CT) of the chest revealed the cause and the diagnosis of CLO was made. A high index of suspicion is needed when clinical and radiological findings are present in a newborn with respiratory distress and recurrent pneumothoraces to ensure prompt diagnosis and optimal, timely treatment.

## Introduction

Congenital lobar overinflation (CLO), previously known as congenital lobar emphysema, is a rare neonatal disease, with a prevalence of 1 in 20,000-30,000 [1-3]. It is a developmental anomaly of the lung [2,4]. Males are more frequently affected than females (3:1) [1,5]. In 50% of cases, the cause is unknown. Otherwise, the most common cause, accounting for 25% of cases, is a congenital defect of bronchial cartilage (e.g., malacia, stenosis), and the other 25% are due to a bronchial obstruction (e.g., mucous plugging, redundant mucosal folds/septa, cardiopulmonary vascular anomalies, intrathoracic masses) [1]. A characteristic finding is progressive pulmonary hyperinflation of one or more lobes [1,5]. Hyperinflation is a result of a one-way valve mechanism of the collapsed airway causing air trapping [5]. Alveolar walls are not destroyed in the process; therefore, it is not true emphysema [1,5]. It leads to compression atelectasis of the remaining lung tissue, with a decrease in ventilated lung tissue, mediastinal shift, and increased intrathoracic pressure. This leads to diminished respiratory reserve and ventilation/perfusion mismatch, resulting in hypoxia [6]. Diagnosis is based on clinical and radiological findings. Prompt diagnosis is essential for optimal treatment [1]. This report presents the case of a newborn twin boy with respiratory distress after birth and recurrent pneumothoraces.

## Case presentation

Informed consent was taken from the parents with full confidentiality maintained regarding the identity of the patient and family.

A newborn boy was born as a first twin at the 26th gestation week in 2006 after his mother was admitted for a sudden increase in uterine contractions, increasing from five per day one day earlier, to more than 30 per day on the day of admission. Magnesium sulfate intravenous was administered, after which the contractions subsided, but then the membranes ruptured. Cesarean section was performed. Prenatal ultrasound scans did not detect any congenital anomalies. Birth weight was 840 g, birth length 32 cm and head circumference 25 cm. Apgar score after one minute was 5, after five minutes was 7, and after 10 minutes was 9. After birth, he was intubated and transferred to the department of pediatric intensive care, because of respiratory distress and prematurity. His vital signs were: blood pressure 45/26 mmHg, heart rate 143/min, respiratory rate 51/min. He was ventilated by synchronized intermittent-mandatory ventilation (SIMV) with positive end-expiratory pressure (PEEP) 5, peak inspiratory pressure (PIP) 21, and inspired oxygen percentage (FiO2) 30%, upon which his oxygen saturation was 100%. On physical examination, the chest was symmetrical, subcostal retractions were not seen; auscultation of the lungs revealed inspiratory and expiratory crackles. The rest of his examination was unremarkable. Due to the clinical and radiological signs of respiratory distress syndrome and hyposurfactosis, he received surfactan within the first day of life. Circulatory support with vasoactive amines was provided because of hypotension

**Figure 1:**
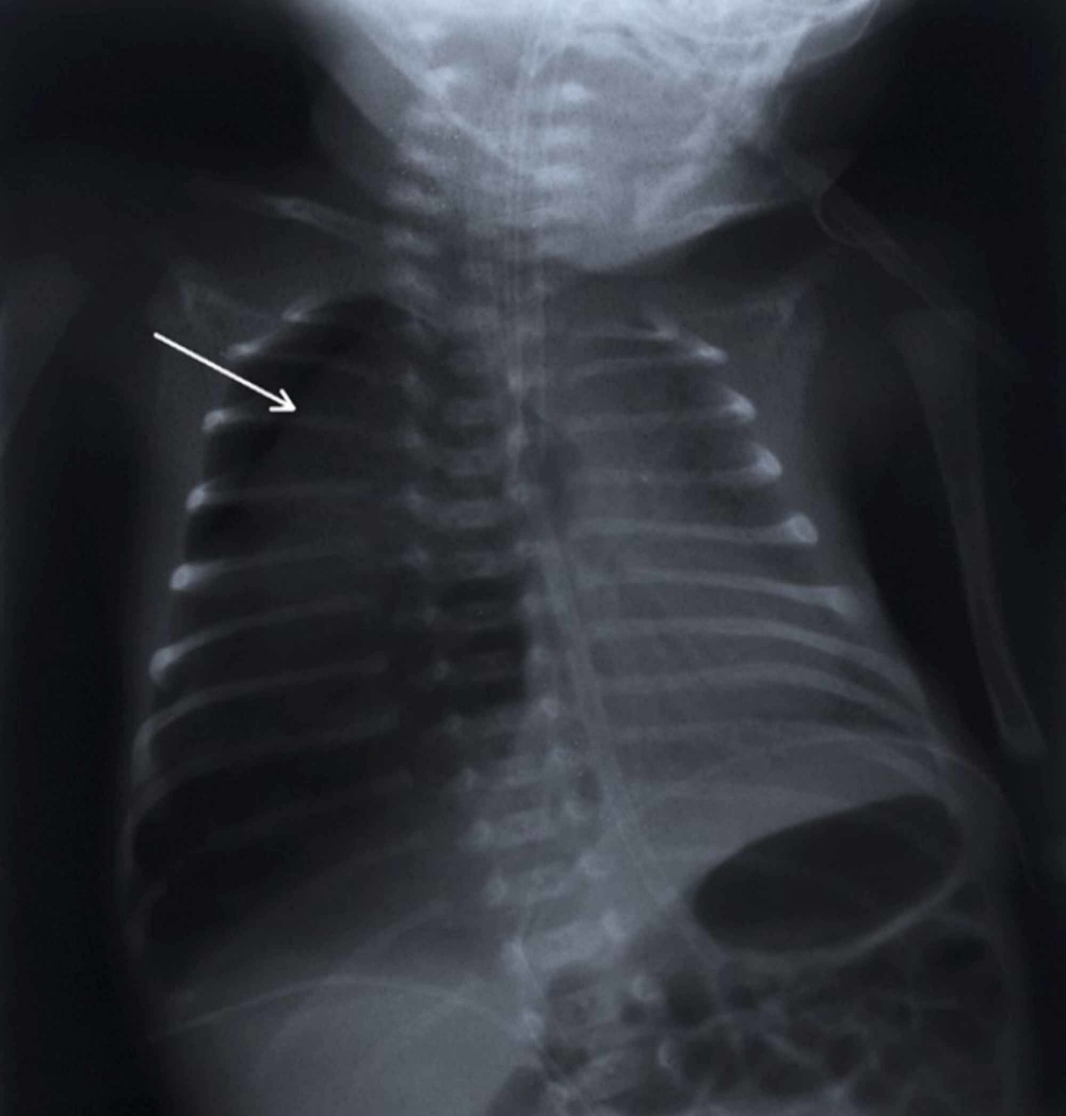
Chest radiograph, posterior-anterior view, performed after birth showing a visceral pleural line (arrow) and no lung markings in pneumothorax on the right side. Mediastinal shift to the left with compression atelectasis on the left side is present

**Figure 2:**
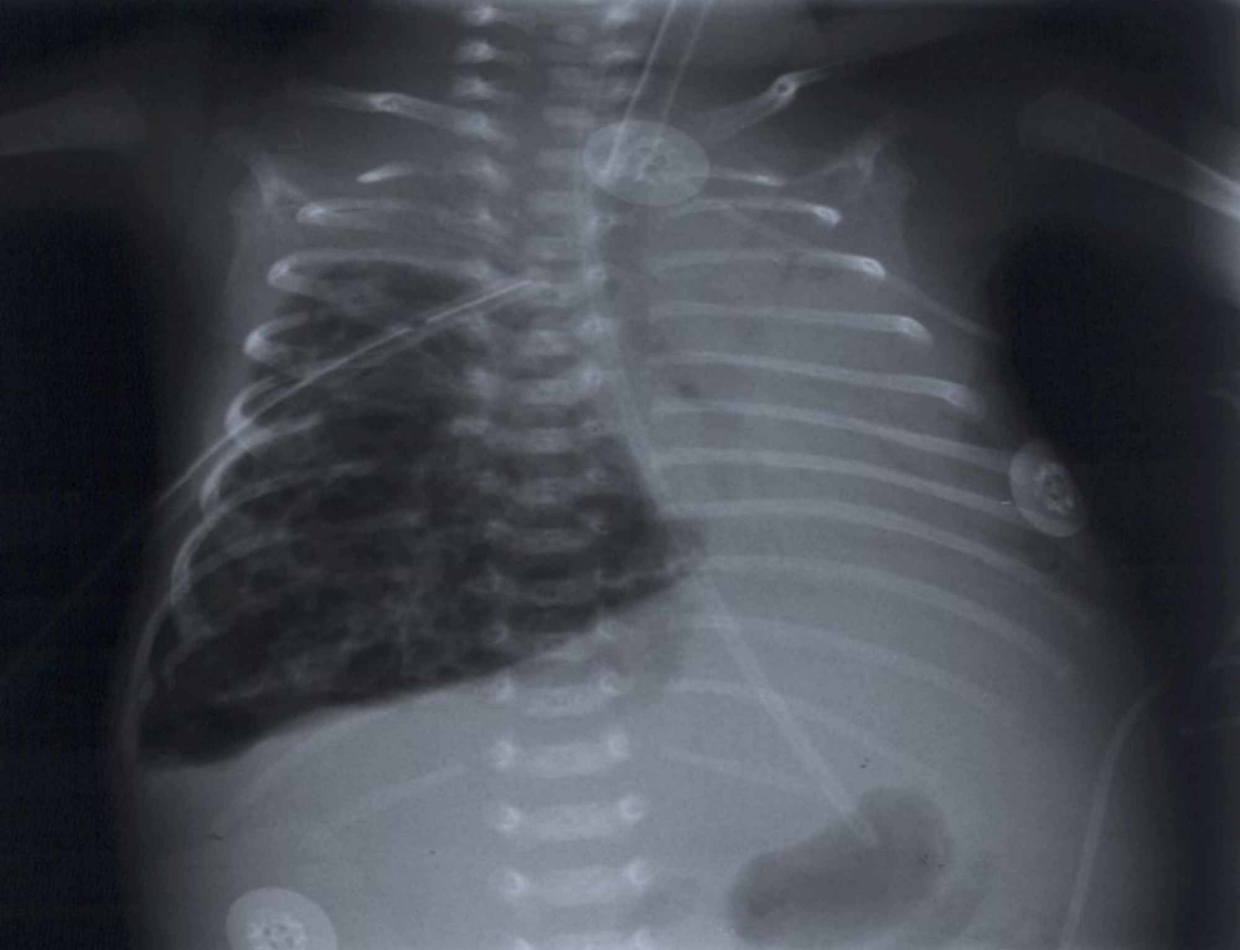
Chest radiograph, posterior-anterior view, performed four days after birth, showing hyperinflation of the right middle lobe. The mediastinal shift to the left and atelectasis of the other lobes are present

**Figure 3:**
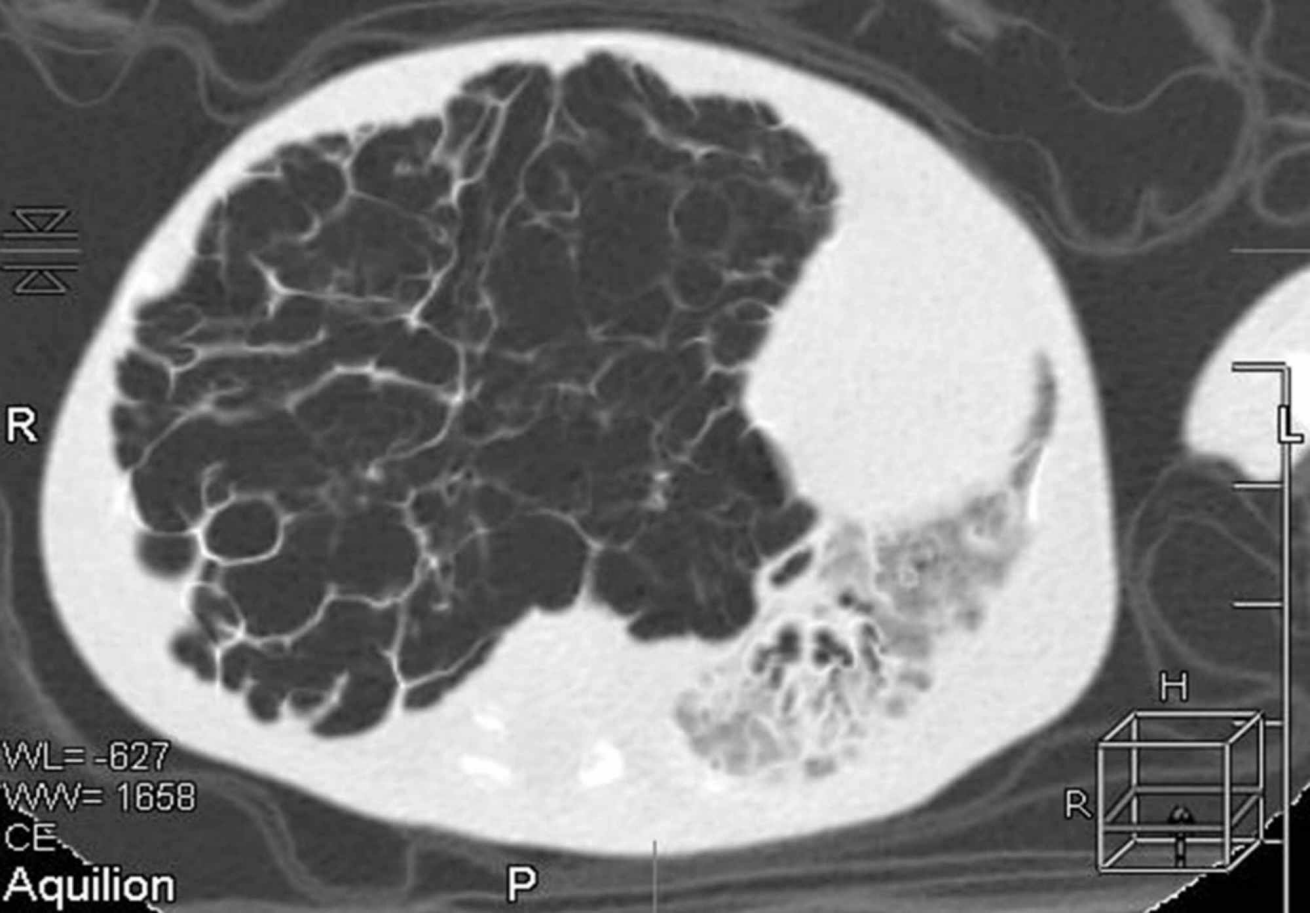
Chest computed tomography (CT), axial view, showing hyperinflation of the right middle lobe, mediastinal shift to the left, and remaining atelectatic tissue

**Figure 4:**
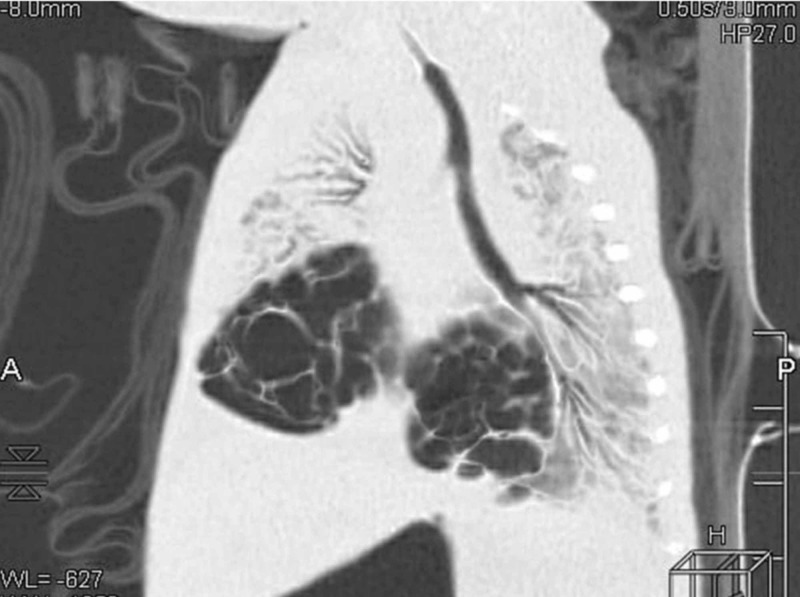
Chest computed tomography (CT), sagittal view, showing hyperinflation of the right middle lobe and atelectatic remaining lobes

On that day, there was a sudden deterioration which was caused by a pneumothorax on the right side of the lungs, detected with a chest radiograph; a chest drain was inserted (Figure 1). Control chest radiograph revealed a persisting difference in aeration of the left and right side of the lungs, which progressed in the next days and weeks even though the chest drain was in place. During the following days, the saturation was unsatisfactory, although he was on SIMV. The boy was cyanotic and SpO2 was often falling below 75%. Pneumothorax was repeatedly observed, even though the chest drains were reinserted and repositioned. On the chest radiograph, lobar hyperinflation of the right middle lobe with mediastinal shift to the left and concurrent atelectasis of the other lobes was observed (Figure 2). High-frequency ventilation was then instituted, after which the boy stabilized and computed tomography (CT) of the chest was performed. It showed hyperinflation of the right middle lobe, which caused the atelectasis of the remaining lung tissue (Figures 3-4). The findings were consistent with CLO. Right middle lobectomy was performed. He was discharged in a stable condition.

Thirteen years after the surgery, the boy has no respiratory problems, but he has a cognitive delay owing to the cerebral hypoxia suffered in the neonatal period.

## Discussion

CLO is a rare neonatal developmental anomaly [2]. It leads to compression atelectasis of the remaining lung tissue, with a decrease in ventilated lung tissue, mediastinal shift, and increased intrathoracic pressure. This leads to diminished respiratory reserve and ventilation/perfusion mismatch, resulting in hypoxia [6]. Approximately 50% of patients are symptomatic in the early neonatal period, with the clinical presentation of respiratory distress [4-5]. Between 20%-30% of patients are symptomatic at birth, and others become symptomatic within six months [4]. The severity of the disease depends on the size of the affected lobe, the surrounding lung tissue compression, and the extension of mediastinal shift with possible venous return impairment [2]. Tachypnea and increased respiratory effort are typically present in infants. They can often become cyanotic [1,4]. Findings on physical examination include retractions, hypersonic percussion, and diminished breath sounds on the affected side [4]. Clinically, the affected children often deteriorate after intubation and use of mechanical SIMV.

Diagnosis can often be established by characteristic signs on chest radiograph; therefore, a chest radiograph is the first step to evaluate the patient with respiratory distress [2,7]. Typical findings on the chest radiograph are distension and unilateral hyperlucency of the affected lobe, mediastinal shift as well as compression and atelectasis of the adjacent and contralateral lung lobes [2,4]. Hyperinflation can cause the diaphragm to look flat. A chest radiograph performed immediately after birth can at first show diminished lucency of the affected lobe, because of retained fetal fluid in the lung, and this can lead to misdiagnosis [2]. When fluid is absorbed in a few days, air fills the lobe, and progressive hyperinflation occurs [2,4]. Differential diagnosis of CLO varies, depending on the findings and the timing of the examination, and includes pneumothorax, atelectasis, congenital adenomatoid malformation, hypoplasia of the lung with contralateral lung hyperinflation, pneumatocele, and diaphragmatic hernia [2]. CLO is often confused with pneumothorax, where on the chest radiograph broncho vascular markings in the radiolucent area are not seen [8]. On the other hand, pneumothorax can be seen concomitantly with CLO, as the hyperinflated lung lobe ruptures. Insertion of the chest drain due to pneumothorax only partially alleviates the problem, as the hyperinflated lobe persists and can expand even further, as long as the intubated child remains on SIMV [6].

CT of the chest remains the main diagnostic method for CLO, and can in some cases reveal the cause and level of airway obstruction [2,8]. To diagnose CLO, a high level of clinical suspicion is needed [6]. In our case, the suggestion that the patient might have CLO was given on the basis of the clinical information (recurrent pneumothoraces in a neonate). Chest CT was performed and it showed a narrowing of the bronchus to the middle right pulmonary lobe. There was excessive inflation of the right middle lung lobe, which caused atelectasis of the remaining healthy lung parenchyma. According to the literature, the right middle lobe of the lung is affected in approximately 30% of cases; left upper lobe in approximately 40%, and right upper lobe in 20% [9]. Lower lobes are involved in less than 1% of all cases [5].

In twin pregnancies, prenatal ultrasonography for scanning of fetal anomalies is preferably performed between 18 and 20 weeks gestation [10]. Prenatal ultrasonography can sometimes detect cystic pulmonary lesions, where CLO presents as a cyst or with increased echogenicity [2,11]. During pregnancy, CLO may vary in size or even disappear; therefore, it can not always be detected on prenatal ultrasound [2]. In our case, there were no abnormal prenatal ultrasound findings.

As treatment with positive pressure ventilation can increase lobar hyperinflation, it is better to use low-pressure, high-frequency ventilation, or pressure-regulated volume-controlled ventilation instead [3]. Selective intubation of the unaffected lung is another possible approach but is difficult to achieve. Severe neonatal respiratory distress may need emergent surgical treatment, where lobectomy of the affected lobe is performed [5,7].

## Conclusions

Recurrent pneumothoraces and worsening respiratory distress in a neonate that was intubated and ventilated by SIMV can suggest the diagnosis of CLO. If specific radiographic findings are present and recognized, the chest radiograph can be diagnostic; otherwise, CT of the chest is the method of choice. Chest radiograph performed early after birth can lead to misdiagnosis because of the presence of the fetal fluid, and the typical picture of a hyperinflated lobe is not yet present. Despite that, we suggest that even if there are no specific findings on the initial chest radiograph, the diagnosis of CLO should not be excluded.
